# Central Nodes in Protein Interaction Networks Drive Critical Functions in Transforming Growth Factor Beta-1 Stimulated Kidney Cells

**DOI:** 10.22074/cellj.2016.4718

**Published:** 2016-09-26

**Authors:** Reyhaneh Rabieian, Maryam Abedi, Yousof Gheisari

**Affiliations:** 1 Department of Genetics and Molecular Biology, Isfahan University of Medical Sciences, Isfahan, Iran; 2 Regenerative Medicine Lab, Isfahan Kidney Diseases Research Center, Isfahan University of Medical Sciences, Isfahan, Iran

**Keywords:** Chronic Kidney Disease, Microarray Analysis, Protein Interaction Maps, Systems Biology, Transforming Growth Factor Beta-1

## Abstract

**Objective:**

Despite the huge efforts, chronic kidney disease (CKD) remains as an unsolved problem in medicine. Many studies have shown a central role for transforming growth factor beta-1 (TGFβ-1) and its downstream signaling cascades in the pathogenesis of CKD. In this study, we have reanalyzed a microarray dataset to recognize critical signaling pathways controlled by TGFβ-1.

**Materials and Methods:**

This study is a bioinformatics reanalysis for a microarray data. The GSE23338 dataset was downloaded from the gene expression omnibus (GEO) database which assesses the mRNA expression profile of TGFβ-1 treated human kidney cells after 24 and 48 hours incubation. The protein interaction networks for differentially expressed (DE) genes in both time points were constructed and enriched. In addition, by network topology analysis, genes with high centrality were identified and then pathway enrichment analysis was performed with either the total network genes or with the central nodes.

**Results:**

We found 110 and 170 genes differentially expressed in the time points 24 and 48 hours, respectively. As the genes in each time point had few interactions, the networks were enriched by adding previously known genes interacting with the differentially expressed ones. In terms of degree, betweenness, and closeness centrality parameters 62 and 60 nodes were considered to be central in the enriched networks of 24 hours and 48 hours treatment, respectively. Pathway enrichment analysis with the central nodes was more informative than those with all network nodes or even initial DE genes, revealing key signaling pathways.

**Conclusion:**

We here introduced a method for the analysis of microarray data that integrates the expression pattern of genes with their topological properties in protein interaction networks. This holistic novel approach allows extracting knowledge from raw bulk *omics* data.

## Introduction

Chronic kidney disease (CKD) is a public health problem and a leading cause of death. Despite using current therapies to slow progression of CKD, respective patients are still reaching the end stage renal disease (ESRD) at alarming proportions (1). The histological feature of this debilitating disorder is excessive deposition of extra-cellular matrix (ECM) defined as renal fibrosis. Recent studies declared that transforming growth factor beta-1 (TGFβ-1) is the major driver of fibrosis in kidney, stimulating a variety of signaling pathways related to deposition of ECM components (2). In spite of enormous researches on the role of TGFβ-1 and downstream elements in the progression of CKD (3, 4), few studies have employed holistic and computational methods for investigation of kidney disorders. Among these studies, there is an elegant report presented by Jin et al. (5) who employed gene regulatory network concepts to analyze high-throughput gene expression data. They could predict and experimentally validate HIPK2 as a potential drug target in HIV-associated nephropathy.

Here, we propose a holistic approach to investigate the molecular interactions and signaling pathways in response to TGFβ-1 stimulation in human kidney cells. A microarray dataset has been generated by Walsh et al. (6) that examines the expression profile of human tubular epithelial cells before and after treatment with TGFβ-1 for 24 and 48 hours. However, they only focused on the few top differentially expressed (DE) genes including *GREM1, JAG1* and *HES1*. They identified Notch signaling as a critical pathway in diabetic nephropathy. In the current study, we introduced a new method for the analysis of the same microarray dataset that integrated the expression pattern of genes with their topological location in the gene interaction network. Using this strategy, we could infer more informative signaling pathways related to TGFβ-1 stimulation. This approach could also be employed for other large data to improve our understanding of biological processes by extracting remarkable concepts from bulk *omics* data.

## Materials and Methods

### Microarray data

This study is a bioinformatics analysis of GSE23338 dataset, originally generated by Walsh et al. (6). mRNA expression profile was downloaded from the Gene Expression Omnibus (GEO) database (7). In this microarray experiment, transcriptional response of human proximal tubule epithelial cells (HK-2) to TGFβ-1 stimulation after 24 and 48 hours was assessed. Using GEO2R tool of GEO, the TGFβ-1 treated cells (24 or 48 hours) were compared to untreated HK-2 cells. Benjamini-Hochberg false discovery rate method was applied for P value adjustment. Genes with adjusted P≤0.05 were considered as differentially expressed.

### Protein-protein interaction network

Using CluePedia plugin (8) of the Cytoscape software version 3.1.0 (9), a protein-protein interaction (PPI) network was constructed for the DE genes in time point of 24 hours or 48 hours. Topology of networks was analyzed by the NetworkAnalyzer tool of Cytoscape software.

### Pathway enrichment analysis

Pathway enrichment analysis for DE genes was carried out using ClueGO plugin (10) of Cytoscape. In this analysis, KEGG and Reactome databases were chosen for retrieving data and network specificity was adjusted to medium. Bonferroni step down was applied for P value adjustment and pathways with adjusted P≤0.05 were chosen.

## Results

**Fig.1:**
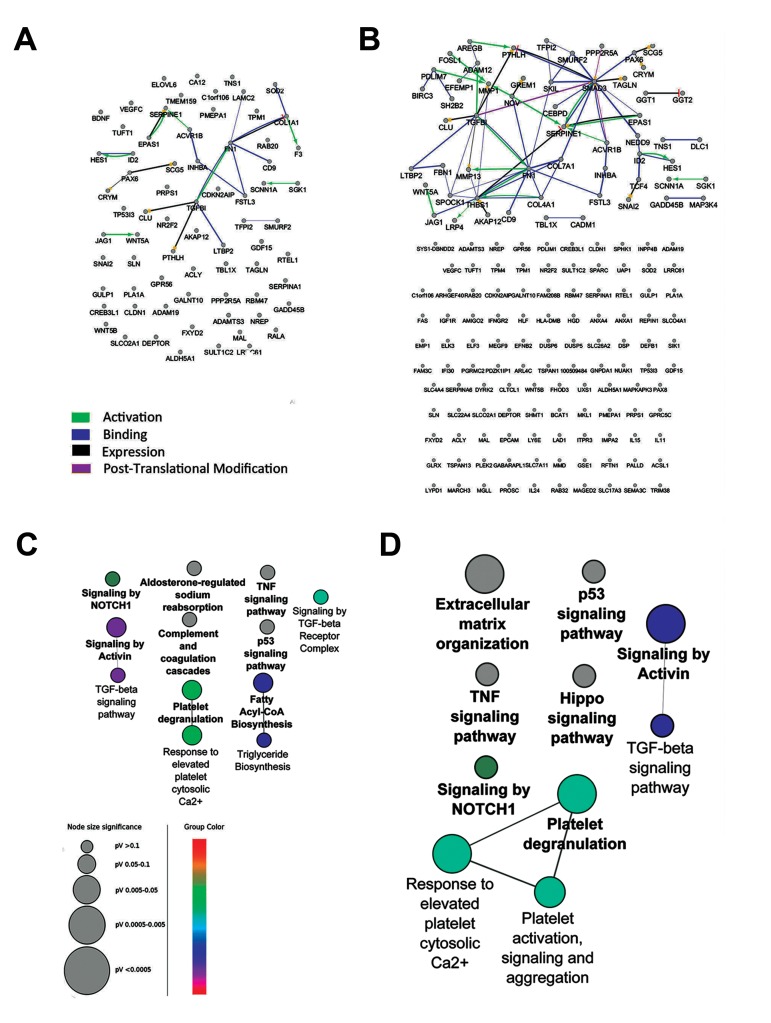
Interaction networks of the DE genes in the microarray dataset were poor and few signaling pathways were enriched. The expression profiles of human kidney cells treated with TGFβ-1 for 24 or 48 hours were compared to untreated cells. The interaction networks of the differentially expressed genes in the time points of A. 24 hours and B. 48 hours have few edges. In addition, pathway enrichment analysis of these genes in C. 24 hours and D. 48 hours could not detect key signaling pathways. Pathways with adjusted P≤0.05 are shown. Color represents the gene ontology (GO) term level. TGFβ-1; Transforming growth factor Beta-1 and DE; Differentially expressed.

In this study, we reanalyzed the GSE23338 microarray dataset assessing mRNA expression profile of HK-2 cells after 24 and 48 hours of treatment with TGFβ-1. Analysis by GEO2R revealed that 110 genes after 24 hours and 170 genes after 48 hours were differentially expressed with adjusted P≤0.05 (Table 1). To investigate the interaction between variably expressed genes, a network was constructed for each time point. Although different kind of interactions (activation, post-translational modification, expression and binding) were allowed to be shown, unexpectedly, few interactions were appeared in both networks (Fig .1A, B). To infer pathways related to the DE genes and understand the down-stream processes controlled by TGFβ-1, pathway enrichment analysis was performed, showing only 12 pathways for 24 hours (Fig .1C) and 10 pathways for 48 hours treatments (Fig .1D), with few connections between the signaling pathways.

**Fig.2:**
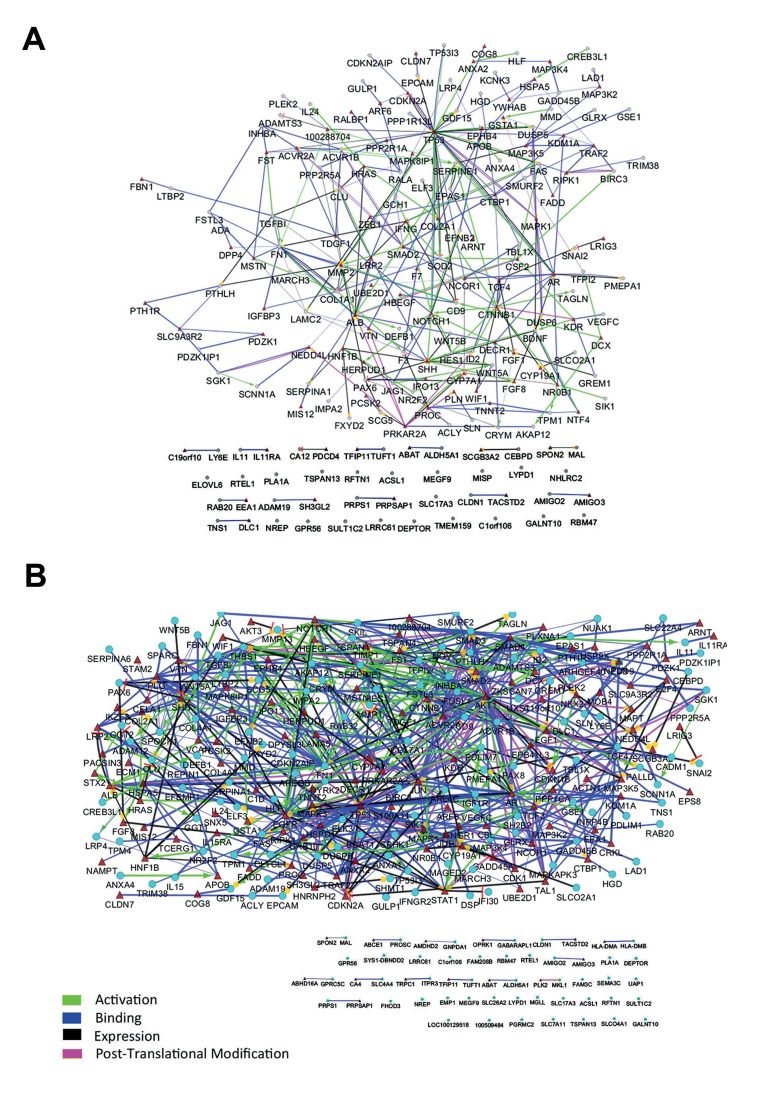
Enrichment of the protein-protein interaction (PPI) network is an efficient method to predict the missed interacting nodes. The networks of A. 24 hours and B. 48 hours treatment were enriched. The selected nodes from microarray experiment are depicted with ellipse and enriched nodes with triangle.

**Fig.3:**
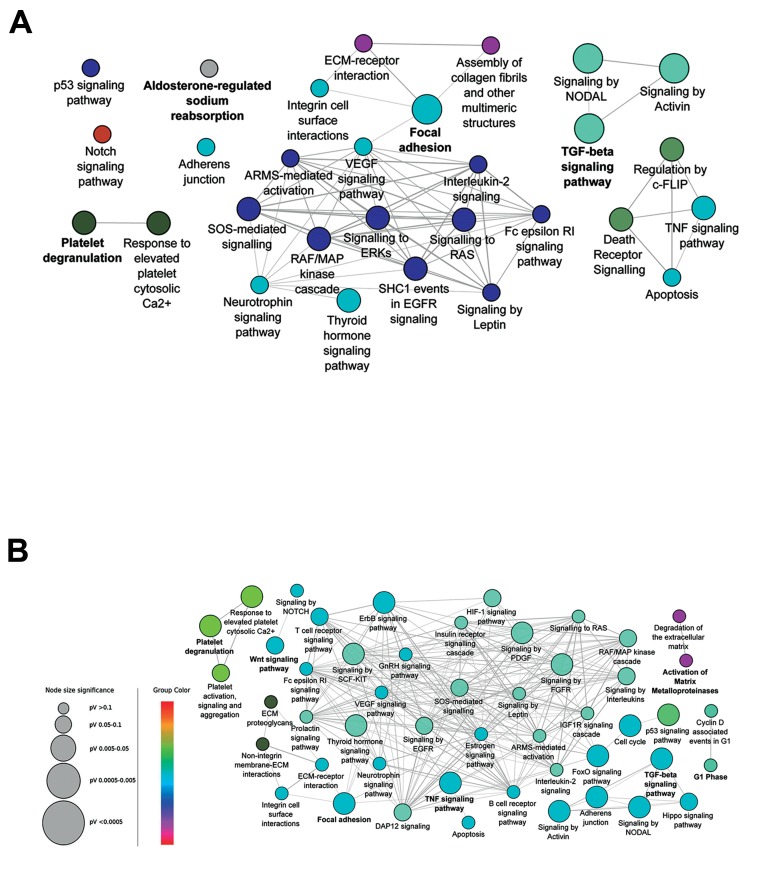
Selection of central nodes for pathway enrichment analysis can detect critical signaling pathways. In the enriched protein-protein interaction (PPI) networks, 62 genes for 24 hours treatment network and 60 genes for 48 hours treatment network were chosen as nodes with high cen trality. These central nodes are related to 29 and 49 highly c onnected pathways in A. 24 hours and B. 48 hours, respectively. Pathways with adjusted P≤0.05 are shown. Color represents the gene ontology (GO) term level.

**Fig.4:**
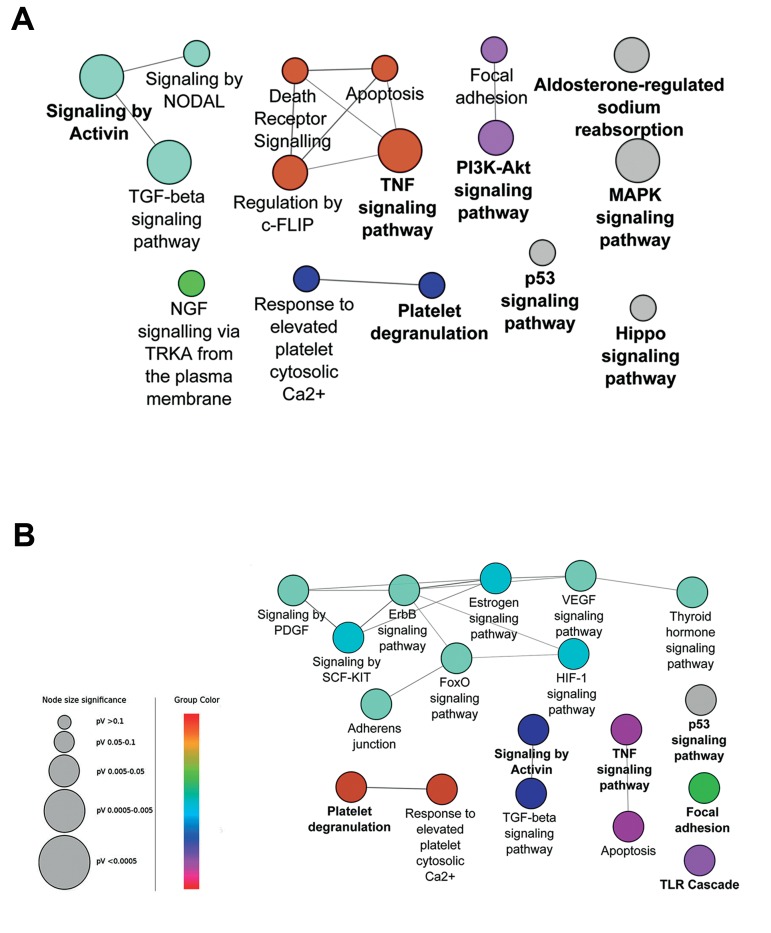
Pathway enrichment analysis with total genes in the enriched network is not informative. Pathway enrichment analysis with all 199 genes in 24 hours, or 301 genes in 48 hours treatment in enriched PPI networks only demonstrated A. 16 or B. 18 poorly inter-connected pathways, respectively. Pathways with adjusted P≤0.05 are shown. Color represents the gene ontology (GO) term level.

The scarcity of interactions in PPI and pathway networks was not unexpected, as they were derived from mRNA microarray data which can only detect genes with altered mRNA level, thus regulated genes at other levels were missed. Hence, to predict other role players, we enriched both PPI networks by adding one interacting node for each gene. This resulted in expansion from 110 to 199 nodes for 24 hours (Fig .2A) and from 170 to 301 nodes for 48 hours treatment (Fig .2B). PPI networks were reconstructed with the same parameters applied initially. To determine the most central genes in these enriched networks, their topology was assessed by graph theory measures such as degree, betweenness centrality, and closeness centrality. In each network, the genes were sorted based on each of these features. Then, the top 20% genes in 24 hours treatment and 15% genes with higher rank in 48 hours were chosen. Because of overlapping nodes between the above three centrality parameters, a total of 62 genes in time point of 24 hours (Table 2) and 60 genes in time point of 48 hours (Table 3) were finally selected. Again, pathway enrichment analysis was performed with either the central genes or the total genes in these two enriched networks. The central genes in time points 24 and 48 hours networks were related to 29 and 49 pathways, respectively (Fig .3). These pathways were strongly related to CKD and formed a deeply connected network in both time points. Interestingly, pathway enrichment analysis with the total enriched networks genes, only determined 16 and 18 pathways for time points of 24 and 48 hours, respectively. These pathways were less inter-connected compared to those derived from the central genes (Fig .4).

Pathway enrichment analysis with the central genes predicted Notch, TNF, P53, Activin and TGFβ signaling as well as platelet-related pathways, affected after TGFβ-1 treatment in both 24 and 48 hours. However, Hippo, PDGF and FGFR signaling pathways were enriched only in the second time point.

**Table 1:** Differentially expressed genes in time 24 hours and 48 hours with adjusted P≤0.05. The genes are sorted by log2 of fold change (LogFC)


Time 24			Time 48		
Genes	adj.P.Val	logFC	Genes	adj.P.Val	logFC

*GDF15*	0.012817	-4.03492	*GDF15*	0.004294	-3.77276
*CRYM*	0.046546	-3.35307	*CRYM*	0.020195	-3.74094
*SCNN1A*	0.012817	-3.19552	*CD9*	0.000557	-3.3273
*CD9*	0.003455	-2.96886	*SCNN1A*	0.006484	-2.86473
*RBM47*	0.012817	-2.96538	*RBM47*	0.010066	-2.73215
*MAL*	0.012817	-2.6579	*MAL*	0.007941	-2.72193
*HLF*	0.033274	-2.44538	*AREG*	0.014332	-2.71598
*DEPTOR*	0.011983	-2.38064	*HLF*	0.021497	-2.52256
*IMPA2*	0.002857	-2.22728	*PLA1A*	0.007423	-2.46499
*RTEL1*	0.003588	-2.11992	*PDZK1IP1*	0.026161	-2.45799
*MEGF9*	0.03429	-2.04315	*DUSP5*	0.005251	-2.37922
*GSE1*	0.011894	-2.04015	*ACSL1*	0.003583	-2.36964
*ELOVL6*	0.004534	-2.02884	*DEPTOR*	0.014818	-2.23285
*BIRC3*	0.012817	-1.98537	*DEFB1*	0.001178	-2.1258
*SLC17A3*	0.006063	-1.96502	*IMPA2*	0.001964	-2.11942
*SULT1C2*	0.045879	-1.93073	*HLA-DMB*	0.036113	-2.11004
*DUSP6*	0.018789	-1.93001	*FXYD2*	0.002471	-2.09686
*CEBPD*	0.015951	-1.89181	*RTEL1*	0.003148	-1.99502
*DEFB1*	0.003455	-1.87388	*CLDN1*	0.002102	-1.9428
*ACSL1*	0.003455	-1.84878	*BIRC3*	0.008587	-1.93307
*PLA1A*	0.030906	-1.79724	*SULT1C2*	0.028474	-1.89456
*DUSP5*	0.011894	-1.78577	*FAS*	0.040775	-1.84699
*CA12*	0.011983	-1.70822	*CEBPD*	0.014469	-1.81201
*CLDN1*	0.006732	-1.69617	*SLC17A3*	0.010066	-1.78837
*PDZK1IP1*	0.031449	-1.66723	*LY6E*	0.003332	-1.70064
*ADAMTS3*	0.009793	-1.64873	*SERPINA1*	0.021497	-1.68148
*CDKN2AIP*	0.047829	-1.62696	*SLCO4A1*	0.03808	-1.67053
*GULP1*	0.049153	-1.55674	*SOD2*	0.003686	-1.65771
*ACVR1B*	0.019538	-1.47953	*TSPAN1*	0.011747	-1.65484
*ID2*	0.018571	-1.45204	*PLIN2*	0.026161	-1.62099
*EPAS1*	0.049153	-1.42294	*MEGF9*	0.024224	-1.61932
*SOD2*	0.016073	-1.41158	*RAB20*	0.026161	-1.59433
*ANXA4*	0.047613	-1.37096	*CLU*	0.002471	-1.54936
*RAB20*	0.015265	-1.34593	*SLC4A4*	0.03487	-1.50061
*MMD*	0.030004	-1.33753	*GULP1*	0.047026	-1.46306
*CLU*	0.01997	-1.32415	*EPAS1*	0.038561	-1.42677
*BDNF*	0.018571	-1.26903	*ACVR1B*	0.013621	-1.3911
*EPCAM*	0.015265	-1.26628	*GPRC5C*	0.026161	-1.34555
*NR2F2*	0.044918	-1.26334	*GSE1*	0.041643	-1.32532
*TMEM159*	0.047829	-1.25784	*LRRC61*	0.020785	-1.32277
*FAS*	0.019538	-1.23999	*ANXA4*	0.038789	-1.31199
*LY6E*	0.014942	-1.20673	*CDKN2AIP*	0.03949	-1.30584
*LRRC61*	0.033972	-1.17462	*MMD*	0.021485	-1.29784
*PPP2R5A*	0.023781	-1.16917	*PPP2R5A*	0.019989	-1.25554
*SERPINA1*	0.039821	-1.09323	*NR2F2*	0.012081	-1.22902
*IL24*	0.011983	-1.09102	*GLRX*	0.035692	-1.22902
*HGD*	0.019538	-1.08015	*SERPINA6*	0.00653	-1.22661
*ELF3*	0.026977	-1.07437	*EMP1*	0.030041	-1.22491
*GCH1*	0.032261	-1.0672	*MAPKAPK3*	0.037211	-1.20559
*ALDH5A1*	0.030004	-1.05748	*IFI30*	0.039032	-1.1775
*FXYD2*	0.020961	-1.02587	*EPCAM*	0.014332	-1.17347
*TRIM38*	0.043165	-0.92721	*SYS1-DBNDD2*	0.039499	-1.16256
*NHLRC2*	0.018571	-0.92091	*ADAMTS3*	0.014586	-1.12871
*TBL1X*	0.040887	-0.88595	*SHMT1*	0.036579	-1.12397
*LAD1*	0.04193	-0.87726	*GGT2*	0.007492	-1.10696
*GLRX*	0.035216	-0.87251	*LAD1*	0.014332	-1.09515
*TPM1*	0.030916	0.782848	*FOSL1*	0.023626	-1.08872
*AMIGO2*	0.032261	0.803279	*ELF3*	0.022045	-1.078
*MISP*	0.030916	0.808838	*ID2*	0.03219	-1.07757
*ACLY*	0.030778	0.809032	*SMAD3*	0.042933	-1.05481
*FN1*	0.03429	0.860918	*IL24*	0.030041	-1.03178
*LYPD1*	0.046955	0.922314	*SH2B2*	0.020195	-1.00971
*RALA*	0.030004	0.95394	*DUSP6*	0.038561	-0.98235
*EFNB2*	0.030004	0.9589	*ITPR3*	0.021485	-0.9804
*SMURF2*	0.044772	1.000129	*PDLIM1*	0.044481	-0.96321
*TFPI2*	0.019538	1.042945	*ALDH5A1*	0.019989	-0.95377
*MARCH3*	0.026013	1.048251	*FAM3C*	0.039499	-0.93464
*NREP*	0.031449	1.121914	*REPIN1*	0.038561	-0.9095
*LTBP2*	0.015265	1.133197	*GGT1*	0.036579	-0.893
*PLEK2*	0.025143	1.137329	*ANXA1*	0.03141	-0.8635
*RFTN1*	0.014768	1.141252	*UXS1*	0.037211	-0.78881
*PRPS1*	0.021243	1.212761	*HGD*	0.039499	-0.77866
*ADA*	0.012817	1.214286	*TBL1X*	0.028029	-0.76181
*TNS1*	0.027064	1.276677	*MGLL*	0.039499	-0.75719
*COL1A1*	0.044918	1.349036	*GNPDA1*	0.028029	-0.75096
*LAMC2*	0.015265	1.448205	*PAX8*	0.031546	-0.73263
*CREB3L1*	0.004425	1.453935	*TRIM38*	0.026161	-0.69388
*TSPAN13*	0.030916	1.468138	*PROSC*	0.047627	-0.68991
*F3*	0.049854	1.537792	*TPM1*	0.045542	0.614444
*AKAP12*	0.030004	1.541307	*ARL4C*	0.038561	0.67729
*HES1*	0.015265	1.549119	*IFNGR2*	0.045542	0.695846
*SGK1*	0.006063	1.584326	*RFTN1*	0.037889	0.727815
*PAX6*	0.014768	1.602106	*ACLY*	0.021485	0.74412
*GREM1*	0.004818	1.607941	*EFNB2*	0.026486	0.789092
*PTHLH*	0.018571	1.651867	*CLTCL1*	0.043748	0.805174
*SLN*	0.030916	1.66995	*SMURF2*	0.019989	0.813175
*ADAM19*	0.046955	1.673182	*FAM208B*	0.038561	0.815648
*TUFT1*	0.01997	1.708363	*TPM4*	0.036579	0.816674
*PPP1R13L*	0.044622	1.715701	*PLEK2*	0.040607	0.838742
*VEGFC*	0.006732	1.731189	*FHOD3*	0.043748	0.840283
*GPR56*	0.005222	1.757315	*CADM1*	0.014818	0.842324
*LRP4*	0.006732	1.839036	*DLC1*	0.035692	0.861077
*SIK1*	0.028431	1.847404	*ELK3*	0.037211	0.866603
*C1orf106*	0.014768	1.852771	*AMIGO2*	0.013633	0.891177
*KCNK3*	0.019891	1.928548	*PGRMC2*	0.038561	0.892116
*WNT5B*	0.015265	1.950651	*RAB32*	0.039499	0.911187
*SNAI2*	0.021356	1.996987	*UAP1*	0.02966	0.914231
*GALNT10*	0.022735	2.016561	*SKIL*	0.037889	0.927445
*GADD45B*	0.005222	2.081882	*MAGED2*	0.047466	0.933606
*FSTL3*	0.006871	2.18737	*DYRK2*	0.045542	0.941228
*WNT5A*	0.015265	2.199978	*PALLD*	0.039499	0.960395
*SCG5*	0.006063	2.421762	*MKL1*	0.012081	0.986708
*TGFBI*	0.010068	2.585222	*MARCH3*	0.039989	1.008954
*TP53I3*	0.018571	2.591672	*LTBP2*	0.007423	1.013795
*IL11*	0.006063	2.680544	*GABARAPL1*	0.026161	1.018263
*PMEPA1*	0.002821	2.69133	*TFPI2*	0.045542	1.023787
*TAGLN*	0.015265	2.807473	*NOV*	0.03219	1.037359
*SLCO2A1*	0.002821	2.969782	*NUAK1*	0.010962	1.041704
*INHBA*	0.006732	3.742935	*SLC22A4*	0.021701	1.057375
*JAG1*	0.012993	4.819474	*PDLIM7*	0.036579	1.075928
			*SEMA3C*	0.040214	1.084533
			*PRPS1*	0.018933	1.090259
			*COL4A1*	0.014469	1.103866
			*NREP*	0.013884	1.110733
			*LYPD1*	0.028474	1.112816
			*TCF4*	0.044016	1.140686
			*GADD45B*	0.047627	1.201497
			*INPP4B*	0.003583	1.212552
			*SGK1*	0.010594	1.225169
			*IL15*	0.036579	1.22672
			*MAP3K4*	0.028944	1.263727
			*TUFT1*	0.037211	1.284833
			*SPARC*	0.019989	1.288601
			*COL7A1*	0.00653	1.297757
			*ADAM12*	0.008731	1.356895
			*CREB3L1*	0.003148	1.386727
			*PTHLH*	0.013101	1.415775
			*ADAM19*	0.026161	1.427201
			*IGF1R*	0.047026	1.47119
			*ARHGEF40*	0.01087	1.471459
			*WNT5B*	0.037889	1.474394
			*C1orf106*	0.018696	1.482021
			*FSTL3*	0.010621	1.530293
			*LRP4*	0.019989	1.533742
			*NEDD9*	0.040607	1.541275
			*HES1*	0.019989	1.573046
			*SPOCK1*	0.014586	1.577949
			*TSPAN13*	0.014818	1.599124
			*SPHK1*	0.024113	1.599544
			*THBS1*	0.047872	1.633499
			*BCAT1*	0.003332	1.666823
			*AKAP12*	0.010066	1.677861
			*SLN*	0.015745	1.68979
			*DSP*	0.048348	1.726407
			*FN1*	0.004908	1.832317
			*SCG5*	0.000815	1.864837
			*GPR56*	0.010962	1.900793
			*GALNT10*	0.028612	1.917703
			*PAX6*	0.005251	1.918114
			*GREM1*	0.001644	1.934697
			*SIK1*	0.012081	1.972459
			*TP53I3*	0.035787	1.979721
			*VEGFC*	0.010621	1.991006
			*EFEMP1*	0.007516	2.118009
			*SLC26A2*	0.026357	2.161277
			*FBN1*	0.019989	2.339046
			*WNT5A*	0.001354	2.385963
			*MMP13*	0.024732	2.392553
			*TAGLN*	0.010066	2.43914
			*SNAI2*	0.002471	2.45019
			*PMEPA1*	0.000815	2.475717
			*TNS1*	0.012081	2.514322
			*TGFBI*	0.002471	2.622322
			*IL11*	0.001964	2.698275
			*SLCO2A1*	0.001145	2.774921
			*SLC7A11*	0.00226	3.076526
			*MMP1*	0.021973	3.220728
			*SERPINE1*	0.000815	3.452134
			*INHBA*	0.000815	3.757617
			*JAG1*	0.007516	4.928316


**Table 2:** The top 20% genes with the best rank in degree, betweenness centrality, and closeness centrality parameters in the enriched proteinprotein interaction (PPI) network of time 24 hours


Geans	Degree	Geans	Betweenness	Geans	Closeness

*TP53*	35	*TP53*	0.338181	*TP53*	0.397906
*FN1*	16	*MMP2*	0.171374	*MMP2*	0.38191
*CTNNB1*	15	*ALB*	0.145517	*NOTCH1*	0.361045
*MMP2*	15	*CTNNB1*	0.125341	*ALB*	0.356808
*ALB*	14	*NOTCH1*	0.11946	*AR*	0.35023
*AR*	14	*SERPINE1*	0.100643	*CTNNB1*	0.347032
*NOTCH1*	14	*AR*	0.085536	*SERPINE1*	0.344671
*SHH*	13	*FN1*	0.075905	*SMAD2*	0.334802
*SMAD2*	11	*SHH*	0.069747	*FN1*	0.333333
*SERPINE1*	10	*SMAD2*	0.069443	*ACVR1B*	0.326882
*COL1A1*	9	*PRKAR2A*	0.067309	*ACVR2A*	0.326882
*PRKAR2A*	9	*HSPA5*	0.067049	*SHH*	0.325482
*MAPK1*	9	*MAP3K5*	0.049224	*CD9*	0.324094
*TGFBI*	8	*PTHLH*	0.047677	*MAPK1*	0.319328
*ACVR1B*	8	*HRAS*	0.045799	*NCOR1*	0.316667
*IFNG*	8	*TGFBI*	0.044403	*LAMC2*	0.31405
*TCF4*	8	*HNF1B*	0.040631	*VTN*	0.312115
*ACVR2A*	8	*CDKN2A*	0.039982	*FAS*	0.310838
*FAS*	7	*NCOR1*	0.039374	*TCF4*	0.310204
*BDNF*	7	*PAX6*	0.038418	*SOD2*	0.308943
*CD9*	6	*CD9*	0.038417	*CTBP1*	0.307692
*LAMC2*	6	*TCF4*	0.037912	*PAX6*	0.306452
*CTBP1*	6	*NR0B1*	0.035918	*HES1*	0.306452
*PAX6*	6	*FAS*	0.035234	*HSPA5*	0.305835
*HES1*	6	*MAPK1*	0.031996	*IFNG*	0.305221
*CSF2*	6	*NEDD4L*	0.030261	*KDM1A*	0.305221
*NR0B1*	6	*SLC9A3R2*	0.028821	*TGFBI*	0.304609
*HNF1B*	6	*IFNG*	0.028734	*CSF2*	0.304609
*LRP2*	6	*CSF2*	0.027151	*PRKAR2A*	0.304
*TRAF2*	6	*ANXA2*	0.026874	*DECR1*	0.303393
*RIPK1*	6	*PROC*	0.026277	*PPP2R1A*	0.302187
*NCOR1*	5	*KDR*	0.024832	*DECR1*	0.303393
*VTN*	5	*CTBP1*	0.024626	*PPP2R1A*	0.302187
*SOD2*	5	*APOB*	0.024534	*COL1A1*	0.30099
*HSPA5*	5	*TRAF2*	0.024347	*BDNF*	0.298625
*CDKN2A*	5	*F3*	0.022625	*TDGF1*	0.295146
*HRAS*	5	*BDNF*	0.022597	*F7*	0.294574
*CYP7A1*	5	*LRP2*	0.022317	*NR0B1*	0.293436
*KDR*	5	*COL2A1*	0.022231	*HNF1B*	0.292308
*ID2*	5	*GSTA1*	0.021794	*CDKN2A*	0.291747
*MAP3K5*	5	*VTN*	0.021145	*DUSP5*	0.290631
*CLU*	5	*ARF6*	0.020175	*LRP2*	0.290076
*NEDD4L*	5	*YWHAB*	0.01996	*ANXA2*	0.289524
*FST*	5	*ACVR1B*	0.018667	*F3*	0.288425
*MSTN*	5	*ACVR2A*	0.018667	*PTHLH*	0.287335
*PROC*	5	*RALA*	0.018621	*HRAS*	0.286792


**Table 3:** The top 15% genes with the best rank in degree, betweenness centrality, and closeness centrality parameters in the enriched proteinprotein interaction (PPI) network of time 48 hours


Genes	Degree	Genes	Betweenness	Genes	Closeness

*TP53*	55	*TP53*	0.218425	*JUN*	0.419966
*AKT1*	49	*AKT1*	0.180618	*TP53*	0.419244
*EGFR*	33	*EGFR*	0.129406	*AKT1*	0.415673
*SMAD3*	32	*JUN*	0.121849	*EGFR*	0.403974
*JUN*	32	*SMAD3*	0.091028	*AR*	0.403306
*AR*	28	*ALB*	0.08664	*SMAD3*	0.394184
*FN1*	25	*CTNNB1*	0.077284	*CTNNB1*	0.3904
*THBS1*	24	*AR*	0.063727	*SMAD4*	0.387917
*CTNNB1*	23	*SMAD4*	0.059499	*SERPINE1*	0.387917
*SMAD2*	23	*FN1*	0.056896	*NOTCH1*	0.380655
*SERPINE1*	20	*THBS1*	0.049411	*THBS1*	0.377709
*SMAD4*	20	*SHH*	0.0474	*SMAD2*	0.375963
*NOTCH1*	18	*NOTCH1*	0.044617	*FN1*	0.371951
*ALB*	16	*SERPINE1*	0.041249	*MMP1*	0.369138
*SHH*	16	*STAT1*	0.039746	*MAPK1*	0.365269
*PLG*	15	*HSPA5*	0.037599	*STAT1*	0.364179
*MMP1*	14	*PLG*	0.035914	*MMP13*	0.359882
*TCF4*	13	*TRAF2*	0.035805	*ALB*	0.357247
*TGFBI*	12	*PRKAR2A*	0.03185	*IGF1R*	0.357247
*MAPK1*	12	*SMAD2*	0.028572	*ACVR1B*	0.350575
*ACVR1B*	11	*SLC9A3R2*	0.028142	*ACVR2A*	0.350575
*CSF2*	11	*HSPD1*	0.027604	*CSF2*	0.34907
*PRKAR2A*	11	*HRAS*	0.025499	*KDR*	0.348074
*STAT1*	11	*TCF4*	0.024472	*CDK1*	0.348074
*TRAF2*	11	*PALLD*	0.024455	*CTBP1*	0.347578
*IGF1R*	10	*TGFBI*	0.024427	*PPP2R1A*	0.346591
*CDKN2A*	10	*STX2*	0.024388	*SHH*	0.343662
*MAP3K5*	10	*CDKN2A*	0.022895	*SPOCK1*	0.343662
*ACVR2A*	10	*CD9*	0.021863	*GRB10*	0.343179
*ID2*	9	*NCOR1*	0.021568	*NOV*	0.342216
*MMP13*	9	*MAP3K5*	0.020279	*GSTA1*	0.33936
*SKIL*	9	*HNF1B*	0.020242	*TCF4*	0.338889
*SPOCK1*	9	*SPOCK1*	0.018718	*FAS*	0.336088
*PDLIM7*	9	*CTBP1*	0.018114	*CDKN2A*	0.335626
*KDR*	9	*TPM1*	0.018056	*NCOR1*	0.335626
*LRP2*	9	*PTHLH*	0.017657	*TGFBI*	0.335165
*TCF3*	9	*TBL1X*	0.016927	*VCAN*	0.334705
*NOV*	8	*CSF2*	0.015788	*HSPA5*	0.334247
*PTHLH*	8	*GSTA1*	0.015304	*CLTCL1*	0.333333
*CDKN1B*	8	*KDR*	0.015185	*SKIL*	0.333333
*GADD45A*	8	*MMP13*	0.014604	*PLG*	0.332879
*GRB10*	8	*ANXA2*	0.01411	*PTHLH*	0.332879
*LAMA5*	8	*CLTCL1*	0.013602	*PRKAR2A*	0.332425
*VTN*	8	*MAPK1*	0.012772	*MAP3K5*	0.330623
*CBL*	8	*TCF3*	0.012502	*LAMA5*	0.330176


## Discussion

 In this study, we reanalyzed a microarray dataset to determine gene expression alteration in response to TGFβ-1 in a human kidney cell line. The investigators who originally generated this data emphasized the involvement of Notch signaling pathway based on a few DE genes (6). In contrast, we have constructed PPI networks for DE genes in the time points of 24 and 48 hours treatment. We found that expansion of these networks followed by selection of central nodes for pathway enrichment analysis is an efficient method to recognize key signaling pathways in response to TGFβ-1 stimulation. Our analysis also predicted the potential role of some novel pathways in this *in vitro* model and also pointed out time-dependent activation of particular pathways. Interestingly, the same investigators later repeated the experiment and assessed the mRNA expression profile by RNA-Seq and found that this technique is superior to microarray in identification of the DE genes and altered signaling pathways (11). Noteworthy, the signaling pathways determined by our analysis on the original microarray dataset is similar to the pathways identified with RNA-Seq data. 

An interesting finding in this study was that pathway enrichment analysis with the DE genes in the microarray experiment was not efficient for prediction of key signaling pathways. However, it was expected that all important genes were not regulated at the mRNA level and so they were not detectable by mRNA microarrays. Therefore, to compensate for this limitation, we constructed a PPI network of DE genes and then enriched this network by adding genes that were previously known to be interacting with the initial network nodes. This expanded gene set was more informative for detecting signaling pathways. Indeed, it is perfect to perform multi-level assessments in biological experiments, but for practical reasons it is not commonly feasible. In this case, it is possible to measure changes at one level and then make bioinformatics predictions to fill the gaps at other levels. 

Several previous studies have shown that highly connected nodes (hubs) in the networks, determined by degree parameter, are vital for the organism survival (12). Next studies revealed that essential genes in the network can be determined not only by degree but also by other centrality parameters, such as betweenness or closeness centrality (13,14). Here, we have used a combination of these three network topology parameters to determine the central nodes. Interestingly, pathway enrichment with these central genes was more informative than enrichment with the initial genes or even with the total genes in the expanded PPI networks. This observation is in line with our recent study on diabetic nephropathy showing the central network nodes tend to be present in signaling pathways and cross talks (15). 

In pathway enrichment analysis, Hippo, PDGF, and FGFR signaling pathways were detected only in the second time point, 48 hours treatment. Actually, the initial activation of upstream signaling pathways detected in 24 hours treatment may lead to the expression of genes, related to these three pathways after 48 hours. This finding on time-specific expression of genes underscores the importance of time-course designs for gene expression analysis experiments. 

Most of the predicted pathways in our analysis such as Notch, TNF, P53, and TGFβ signaling have been previously known to be involved in the pathogenesis of CKD (16,19), whereas, for some others, such as platelet degranulation pathway, there is not currently direct experimental proof for participation in renal fibrosis. However, previous experiments have shown megakaryocytes as mediators of fibrosis in a subset of hematologic malignancies, idiopathic pulmonary fibrosis, as well as bone marrow (20,22). The role of megakaryocytes in kidney fibrosis is an interesting topic for future studies. 

## Conclusion

We have here employed a holistic approach to assess the consequences of TGFβ-1 stimulation in kidney cells. Although, high-throughput techniques are frequently applied in biological investigations, data interpretation is yet commonly limited to the assessment of most up or down-regulated factors missing the huge effect of interactions for genes with subtle expression change. Systems biology provides novel concepts and methods to infer the underlying mechanisms of biological phenomena from *omics* raw data and hopefully will bring a higher quality of life to those suffering from chronic diseases. 
